# Free-living amoebae and their role in *Piscirickettsia salmonis* transmission in Chilean Salmon aquaculture: insights from *in vitro* and *in vivo* studies

**DOI:** 10.3389/fmicb.2025.1711258

**Published:** 2026-01-06

**Authors:** Fernando A. Gómez, Bruno Milesi, Sergio H. Marshall

**Affiliations:** Laboratorio de Genética e Inmunología Molecular, Facultad de Ciencias, Instituto de Biología, Pontificia Universidad Católica de Valparaíso, Valparaíso, Chile

**Keywords:** *Piscirickettsia salmonis*, FLA, Infection, *Piscirickettsia*, salmon farming Chile

## Abstract

*Piscirickettsia salmonis* is the causative agent of Rickettsial Septicemia (SRS), a severe disease threatening the sustainability of the growing salmon farming industry in Chile. This pathogen significantly impacts fish throughout their life cycle in the ocean, affecting pathogen-free individuals sourced from certified hatcheries. Systematic outbreaks often lead to catastrophic losses near harvest time, suggesting that primary infections originate from an environmental source that remains largely unidentified. Given the ubiquitous nature of free-living amoebae (FLA), we investigated their potential role as reservoirs and vectors for *P. salmonis*. We focused on *Acanthamoeba castellanii* and *Vannella* sp., two FLA species associated with salmon populations. Using immunofluorescence and quantitative PCR (qPCR), we demonstrated that *P. salmonis* can be internalized, replicate, and be released by both amoeba species. Furthermore, *P. salmonis showed an upregulation on its Dot/Icm* secretion system, essential for intracellular replication, during this process. While *Vannella* sp. exhibited pathogen-induced lysis after 72 h, *A. castellanii* maintained the bacteria more stably. Our findings indicate that *A. castellanii* acts as a reservoir and vector for *P. salmonis*, potentially contributing to the persistence and spread of this pathogen in aquatic environments. This enhanced understanding of the pathogen’s life cycle has implications for developing improved disease control strategies.

## Introduction

*Piscirickettsia salmonis* is a Gram-negative, facultative intracellular bacterium that poses a significant threat to global salmon aquaculture. Responsible for high mortality rates, up to 52.2%, it results in substantial economic losses, particularly in regions like Chile (Rozas and Enríquez, 2014; Servicio Nacional de Pesca y Acuicultura (SERNAPESCA), 2024). Despite advances in understanding its biology, critical knowledge gaps remain regarding its virulence factors, antimicrobial resistance genes, and genomic variability (Islam et al., 2025). The pathogen’s ability to survive within host cells complicates control efforts, as it can infect multiple salmonid species, including *Salmo salar*, *Oncorhynchus mykiss*, and *O. kisutch*. Its persistence mechanisms and transmission dynamics are not fully elucidated, hindering effective prevention strategies. Emerging evidence suggests that *P. salmonis* plays an increasingly prominent role in disease outbreaks, emphasizing the need to understand its lifecycle, reservoirs, and transmission pathways, especially in marine environments. In recent decades, although historically described as an intracellular bacterium, several optimized axenic media have been developed for the *in vitro* growth of *P. salmonis* (Yáñez et al., 2012; Henríquez et al., 2013; Contreras-Lynch et al., 2017). Serial propagation leads to genomic reorganizations, including gene translocations between chromosomes and plasmids, further complicating pathogenicity studies (Valenzuela-Miranda et al., 2020). Additionally, genomic diversification within the genus remains poorly characterized due to limited complete genomes (Schober et al., 2023). Environmental reservoirs of *P. salmonis* remain largely unidentified, although repeated reinfections of farmed salmon suggest persistent, unrecognized sources in coastal waters. Given the bacterium’s exposure to environmental stressors, it likely seeks refuge in niches that facilitate survival, replication, and transmission. Recent studies demonstrate that *P. salmonis* employs complex metabolic pathways in response to environmental challenges (Schober et al., 2023; Aliaga-Tobar et al., 2025).

Free-living amoebae (FLA) are ubiquitous protozoa capable of surviving in a wide range of extreme environmental conditions, including fluctuations in pH, temperature, osmotic pressure, and nutrient availability. These organisms typically alternate between two distinct life stages: (i) Trophozoite, a metabolically active, vegetative state that predominates under favorable environmental conditions and supports binary fission; and (ii) Cyst, a dormant, resistant form induced under adverse conditions to ensure survival. Encystment is triggered by a variety of environmental stressors, including nutritional deprivation (Neff et al., 1964; Pickup et al., 2007; Khan et al., 2015), physicochemical changes (Cordingley et al., 1996; Aksozek et al., 2002; Salah et al., 2009; Coulon et al., 2010; Serrano-Luna et al., 2013), and biological interactions (Denoncourt et al., 2014). Amoebae primarily obtain nutrients through the phagocytosis of bacteria, which are enclosed within phagolysosomes and subjected to oxidative stress, hydrolytic enzymes, acidification, nutrient limitation, and exposure to antimicrobial molecules (Greub and Raoult, 2004; Balczun and Scheid, 2017; Tosetti et al., 2014; Strassmann and Shu, 2017).

While many bacteria are degraded under these hostile conditions, a subset known as amoeba-resisting bacteria (ARB) are capable of surviving and even replicating within amoebae. These bacteria may exploit the intracellular environment for protection and persistence, particularly when amoebae encyst while harboring viable bacterial cells. ARB employ a range of sophisticated strategies to evade amoebic defenses, including inhibition of phagosome–lysosome fusion, modulation of phagosomal pH, disruption of phagosomal membranes, and attenuation of oxidative stress responses. Some ARB translocate from the phagosome to the cytosol or nucleus of the amoebal host, while others remain within modified phagosomes that are permissive to bacterial survival and replication (Strassmann and Shu, 2017). This dynamic interaction underscores the ecological and biomedical significance of FLAs as environmental reservoirs and training grounds for bacterial pathogens, many of which exhibit enhanced resistance to macrophage-mediated killing and antimicrobial treatments. In the context of the One Health framework, free-living amoebas (FLA) are integral components of aquatic ecosystems, interacting with bacteria, fungi, and protists. They serve as ecological regulators but can also act as vectors, fostering bacterial persistence and transmission (Scheid, 2018; Fan et al., 2024). Notably, interactions between *P. salmonis* and FLA, particularly in salmon production environments, remain underexplored. In fact, FLA are common in aquatic environments, where they inhabit biofilms, sediments, and water interfaces, forming trophozoites and cysts that withstand adverse conditions (Fan et al., 2024; Potgieter et al., 2021). Within these microbial niches, FLA may both prey on and harbor bacteria intracellularly, acting as environmental reservoirs or protective hosts (Amissah et al., 2014). These traits make them plausible environmental hosts for fish-pathogenic bacteria in aquaculture systems. In this frame, FLA acts as natural hosts for various aquatic bacteria, including *Legionella pneumophila* and *Vibrio cholerae, which* can live and even multiply within *Acanthamoeba castellanii*, while biofilms and sediments provide additional protection for these associations (Van der Henst et al., 2016; Nisar et al., 2020). In salmon aquaculture, multiple amoebal taxa, including *Vannella*, colonize AGD-affected gills, supporting the ecological plausibility of FLA-bacteria interactions in this system (English et al., 2019). These precedents support the idea that *P. salmonis* could establish comparable interactions with amoebae in marine systems.

Previous studies have shown that *Piscirickettsia salmonis* can persist and express virulence factors inside fish cells (Islam et al., 2025). However, its interaction with protozoan hosts has not been explored. This study provides the first experimental evidence that *P. salmonis* can survive within free-living amoebae under controlled conditions, a mechanism resembling strategies used by *Legionella pneumophila* to persist in aquatic environments (Rowbotham, 1980). In this study, we investigate whether known FLA associated with net-pen environment participate in the life cycle and infectivity of *P. salmonis*. We demonstrate that *P. salmonis* can infect, multiply within, and be maintained by *Acanthamoeba castellanii* and *Vannella* sp., confirming its capacity to utilize protozoa as vectors. During intracellular residence, *P. salmonis* overexpresses genes linked to intracellular multiplication, including components of the Dot/Icm secretion system, crucial for pathogenicity in fish macrophages (Gómez et al., 2013; Mancilla et al., 2018). These findings advance our understanding of *P. salmonis* ecology and highlight potential avenues for controlling its spread, thereby supporting the sustainability of salmon aquaculture in Chile.

## Materials and methods

### *Piscirickettsia salmonis* growth conditions

*Piscirickettsia salmonis* LF-89 strain (ATCC VR-1361) was cultured on sheep blood agar plates supplemented with 0.1% L-cysteine and 1% glucose (Mauel et al., 1999) at 23 °C.

### *Acanthamoeba castellanii* growth conditions

*Acanthamoeba castellanii* (ATCC® 30,234) was routinely maintained at 25 °C in the dark without agitation in ATCC® Medium 712 PYG with additives (ATCC.org). To evaluate the potential for co-culture with *P. salmonis*, the growth of *A. castellanii* was tested in various media: Austral SRS-Broth, MC1, BM3, MM, IFOP, and 712 PYG used as a positive control (Yáñez et al., 2012; Henríquez et al., 2013; Contreras-Lynch et al., 2017). Cultures were incubated at 25 °C for 7 days in the dark without agitation. Subsequently, *A. castellanii* growth was assessed over an additional 7 days in three variations of 712 PYG medium: full-strength (standard), half-strength (712 PYG/2), and the salt buffer component of 712 PYG.

### SHK-1 cell line culture and maintenance

The SHK-1 cell line, derived from head kidney leukocytes of *Salmo salar*, exhibits macrophage-like and fibroblast-like morphology (Dannevig et al., 1995). Cells were maintained at 18 °C in 75 cm^2^ culture flasks using Leibovitz’s L-15 medium (Invitrogen) supplemented with 15% fetal bovine serum (FBS; Gibco BRL). Cells were then seeded into 6-well plates for *A. castellanii* challenge assays.

### Infection kinetics of *Piscirickettsia salmonis* in *Acanthamoeba castellanii*

Trophozoites of *A. castellanii* were cultured in 20 mm glass-bottom Petri dishes (NEST®) at a density of 1 × 10^5 cells per dish in PYG/2 medium. Cultures were incubated overnight at 25 °C to allow adherence. Cells were then challenged with *P. salmonis* at a multiplicity of infection (MOI) of 10 for 2 h at 18 °C under static, dark conditions. After incubation, the medium (2 mL) was gently removed, and cells were washed twice with fresh medium (2 mL) to eliminate non-adherent bacteria and bacteria in suspension. Thirty minutes before fixation, samples were stained with LysoTracker™ Red DND-99 to label acidic compartments. Co-cultures were fixed with 1 mL of ice-cold 100% methanol and permeabilized with 0.1% Triton X-100 in 1 × PBS for 15 min at room temperature. Indirect immunofluorescence staining of *P. salmonis* was performed using the SRS Fluorotest Indirect Kit (Ango). Fixation and immunostaining were carried out at 2, 24, 48, and 168 h post-infection. Samples were visualized with a Leica CTR5000 epifluorescence microscope equipped with Leica Application Suite V4 software.

### Challenge of SHK-1 cells with *Acanthamoeba castellanii* previously infected with *Piscirickettsia salmonis*

Time-course experiments were conducted in which SHK-1 cells at approximately 80% confluence were challenged with *A.* castellanii trophozoites and cysts previously infected with *P. salmonis*. Encystment of *A. castellanii* was induced by incubating infected cultures at 5 °C for 4 h. The progression of infection in SHK-1 cells was monitored by microscopy until the appearance of characteristic cytopathic effects associated with *P. salmonis* infection, which include cell rounding, vacuole formation, and detachment (Rojas et al., 2009).

### Isolation and maintenance of marine amoebae

Marine amoebae were isolated from gill and mucosal samples collected from *Salmo salar* exhibiting signs of amoebic gill disease (AGD) at aquaculture facilities in the Aysén region. Samples were initially cultured in Maltose Yeast Broth (MYB) medium before being plated on MYB agar. Protozoan isolation involved successive transfers and serial dilutions; however, complete separation from bacteria was not achieved. Cultures of the isolated amoebae were maintained in MYB medium diluted to 1/14 of its original concentration with sterile seawater, incubated at 18 °C in the dark under static conditions in 25 mL vented culture flasks with a total volume of 3 mL. To prevent bacterial overgrowth in the medium, cultures were washed with 2 mL of sterile seawater, and the medium was replaced every 2 days or whenever excessive bacterial confluence was observed. Finally, to eliminate and/or inhibit the growth of the associated symbiotic bacteria, we tested increasing antibiotic concentrations ranging from 10 μg/mL to 50 μg/mL of Streptomycin (STR), Gentamicin (GEN), Kanamycin (KAN), Polymyxin B (PMB), Ciprofloxacin (CIP), as well as selected combinations of these compounds. Additionally, Imipenem was evaluated at concentrations ranging from 3 μg/mL to 30 μg/mL.

### Identification of isolated marine amoeba

The isolated marine amoeba was initially identified as *Vannella* sp. based on morphological characteristics following the criteria described by Smirnov et al. (2007). To validate this morphological identification, 87 partial 18S rRNA gene sequences belonging to the *Vannella* genus were retrieved from GenBank (NCBI). These sequences were aligned using multiple and local alignment methods to facilitate the design of universal primers for conventional PCR targeting *Vannella* sp (See Table 1). Total DNA was extracted from 11 different amoeba cultures using the GeneJET Genomic DNA Purification Kit (Thermo Scientific™). Conventional PCR was conducted using generic primers for the SSU rRNA gene, following this protocol: initial denaturation at 95 °C for 5 min; 30 cycles of 95 °C for 30 s, 55 °C for 30 s, and 72 °C for 30 s; with a final extension at 72 °C for 5 min. PCR products were sequenced by Macrogen, Inc. (South Korea). The resulting sequences were analyzed using BioEdit v7.0.5 and MEGA X software (Kumar et al., 2018) and further confirmed via the online BLASTn tool (Altschul et al., 1990; Boratyn et al., 2012).

**Table 1:** Primers used for different PCR and qPCR analyses.

Primer name	Organism	Sequence	Tm	Reference
TaqAcF1	*A. castellanii*	CGACCAGCGATTAGGAGACG	62 °C	Rivière et al. (2006)
TaqAcR1		CCGACGCCAAGGACGAC	60 °C	
DotA(16)-F	*P. salmonis*	TCCCGCCTGTATTGAAGAGAGTG	58,5°C.	Gómez et al. (2013)
DotA(16)-R		CGAAAATCCGACCAATCACAGCA	58,0°C	
DotB(16)-F	*P. salmonis*	CCTTACGGAGAAAGCCTGGGTTA	58,5°C	Gómez et al. (2013)
DotB(16)-R		AATGCCTGCCAAATAATCACCCG	58,3 °C	
DotD(16)-F	*P. salmonis*	TGAAATTCGCCGTGCACAATACC	58,4 °C	Gómez et al. (2013)
DotD(16)-R		GCGGATGCGGATGGATAATTAAT	55,5 °C	
*sdhA*-For	*P. salmonis*	ATTTCTTTGGAGCTACGTGAAG	53,0°C	Flores-Herrera et al. (2018)
*sdhA*-Rev		CCACCCATCATATAATGACAAG	50,9 °C	
RTS1	*P. salmonis*	TGATTTTATTGTTTAGTGAGAATGA	56 °C	Marshall et al. (1998)
RTS4		ATGCACTTATTCACTTGATCATA	56 °C	
VGL	*Vannella* sp.	CCGCGGTAATTCCAGCTCT	59.5°C	This work
VGR		CCTTGTTACGACTTTTGCTTCC	60.1°C	
18sVFor	*Vannella* sp.	GGATGGTTAGCGGTGAAATG	58.4 °C	This work
18sVRev		CATATCCCTGGTCGGCATAG	60.5°C	
S17Vanf	*Vannella* sp.	ACGAGACCTTTACCACAT	51,4 °C	Smirnov et al. (2007)
Ampvf		ACCAGGTCCAGACATTAG	53,8°C	

### Detection of *Piscirickettsia salmonis* in isolated marine amoebae

To detect *P. salmonis* in cultures of the isolated protozoan (which had not been experimentally exposed to *P. salmonis*), total DNA was extracted from 11 high-confluence culture flasks using the GeneJET Genomic DNA Purification Kit (Thermo Scientific™). The DNA served as a template for conventional PCR targeting the ITS region of *P. salmonis*, using primers RTS1 and RTS4 (see Table 1). The PCR protocol was initial denaturation at 95 °C for 5 min; 30 cycles of 95 °C for 30 s, 51 °C for 30 s, and 72 °C for 30 s; with a final extension at 72 °C for 5 min. Additionally, detection of the *sdhA* gene and ITS marker was performed via quantitative PCR (qPCR) using the KAPA SYBR® FAST qPCR Kit (Merck), following the manufacturer’s instructions and using gene-specific primers listed in Table 1. Further validation employed indirect immunofluorescence testing (IFAT). *Vannella* sp. cultures were grown in 6-well plates with 3 mL of sterile seawater until confluence. The supernatant was removed, and each well was fixed with 200 μL of 37% formaldehyde for 20 min. Wells were washed three times with 1 mL of 1 × PBS, then permeabilized with 200 μL of 0.1% Triton X-100 for 20 min. After three additional PBS washes, 200 μL of the oligoclonal reagent (SRS Fluorotest Kit, Ango) was added, and plates were incubated overnight at 4 °C. The wells were then washed three times with 1 mL of washing buffer. Four wells were treated with 300 μL of a 1:200 dilution of Anti-Mouse IgG (H + L) Antibody, Human Serum Adsorbed, and Peroxidase-Labeled (KPL) for 1 h. After washing, these wells were incubated with 300 μL of a 1:1000 dilution of Donkey anti-Goat IgG (H + L) Cross-Adsorbed Secondary Antibody, Alexa Fluor 488 (Invitrogen). The remaining wells followed the manufacturer’s protocol, with a one-hour incubation and subsequent washes. All wells were stored in 200 μL of 1 × PBS at 4 °C until visualization under an inverted microscope (Eclipse Ti-S, Nikon®).

### Infection of isolated marine amoeba with *Piscirickettsia salmonis*

The isolated marine amoeba was infected with *P. salmonis* in 24-well plates containing 2 mL of PYG medium, incubated at 18 °C in darkness without agitation, at an MOI of 1:1 for up to 168 h (7 days). Prior to infection, *P. salmonis* cells were stained with R18 dye (Invitrogen) following the manufacturer’s protocol. The concentrations of both organisms were determined by direct counting: amoebae were quantified via microscopy, and *P. salmonis* concentrations were measured using Petroff-Hausser counting chambers. Amoebae were cultured in 3 mL of seawater until reaching approximately 2–3 × 10^7 cells per well in 6-well culture plates (3.5 cm diameter). Co-incubation with *P. salmonis* at an MOI of 1:1 was performed, with samples collected at 2, 24, 48, and 72 h post-inoculation for DNA and RNA extraction. For the amoeba-bacteria interaction assays, both supernatant and pellet fractions were collected to evaluate extracellular and intracellular *P. salmonis*, respectively. Control assays included *P. salmonis* cultured in seawater and amoebae cultured in their respective media, from which supernatants and pellets were obtained and subsequently combined for further analyses. Supernatant samples were centrifuged at 5,000 rpm for 5 min, and pellets were washed twice with 1 mL of 1 × PBS to remove surface-associated bacteria, followed by centrifugation at 5,000 rpm for 5 min. All experiments were performed in triplicate. Depending on the downstream application, genomic DNA and total RNA were extracted using the Thermo Scientific GeneJET Genomic DNA Purification Kit and the E. Z. N. A.® Total RNA Kit I (Omega Bio-tek), respectively, following the manufacturers’ instructions. Absolute quantification of *P. salmonis* DNA was performed using an internal transcribed spacer (ITS) calibration curve.

### Challenge of *Salmo salar* with marine amoebae infected with *Piscirickettsia salmonis*

For the challenge were used Atlantic salmon (*Salmo salar*) smolts with an average weight of 37,5 g were obtained from Centrovet-Virbac Laboratories. Prior to the trials, 10 fish were randomly sampled and confirmed to be free of *P. salmonis*, *Renibacterium salmoninarum*, and ISAv by qPCR assays. Four 130-L tanks were used, maintained at 14 ± 0.5 °C, with oxygen saturation between 55 and 88%, in closed recirculation systems equipped with automatic control of parameters including temperature, dissolved oxygen, flow rate, and physicochemical factors (nitrite, nitrate, phosphate, ammonium/ammonia, total chlorine, salinity, total hardness, phosphate, and pH). Twenty fish per tank were kept for 4 weeks and underwent a 10-day acclimation period to minimize stress-related confounding factors. A photoperiod of 14 h light and 10 h darkness was maintained, with feeding at 1.5–2.0% of body weight and size.

Four experimental groups were established:

- Group 1 (Tank 1): *P. salmonis* at 1 × 10^7^ bacteria/mL, with 500 μL of 1 × PBS per fish (Infection control).
- Group 2 (Tank 2): 500 units of *Vannella* sp. per liter, previously infected with *P. salmonis*.
- Group 3 (Tank 3): Infected *Vannella* sp. containing 1 × 10^7 *P. salmonis* per liter.
- Group 4 (Tank 7): 500 units of *Vannella* sp. per liter (uninfected control).

For the challenge, fish were sedated with Kalmagin 20% (Centrovet) at 1.5% concentration in the culture water to immobilize them prior to inoculation. Bacterial inoculation was performed via intraperitoneal injection to ensure consistent dosage and inoculum quality. Conversely, inoculation with unchallenged amoebae, amoebae previously infected with *P. salmonis*, and bacteria associated with the marine protozoan was carried out by immersing fish in 40 L tanks containing the respective agents for 3 h. After recovery, fish were returned to the main culture tanks. The formation of experimental groups and inoculation procedures were designated as day 0 of the infection kinetics study. Fish samples were collected on days 0 (inoculation day), 6, 15, 21, and 30. Three fish per group were sampled on each day from both control and challenged groups. The anterior kidney was collected, as this tissue is a major lymphoid organ in teleost fish. Additionally, the liver was selected because it is a key target organ for systemic *P. salmonis* infections. The organs were resuspended in 1 mL of RNAlater™ Stabilization Solution (Thermo Fisher) and stored at −80 °C for subsequent RNA extraction, following the manufacturer’s instructions.

### qPCR and RT-PCR quantification for challenges with amoebae infected with *Piscirickettsia salmonis*

RNA samples were reverse-transcribed into complementary DNA (cDNA) using the RQ1 RNase-Free DNase (Promega) and the iScript™ cDNA Synthesis Kit (Bio-Rad), according to the manufacturers’ instructions. Relative gene expression at each time point was evaluated for selected genes of the Dot/Icm secretion system. The *sdhA* and ITS genes of *P. salmonis* served as reference genes. Amplifications were performed using the KAPA SYBR® FAST qPCR Kit according to the manufacturer’s protocol, with primers specific for each target gene (see Table 1). The thermal cycling protocol included an initial denaturation step at 95 °C for 3 min, followed by 40 cycles of 95 °C for 5 s, primer-specific annealing temperature for 15 s, 60 °C for 3 s, and a melting curve analysis from 60 °C to 95 °C with increments of 0.5 °C every 5 s. All data were analyzed using the CFX Manager™ Software (Bio-Rad). The genes evaluated and primers used in different experiments are detailed in Table 1.

## Results

### *Piscirickettsia salmonis* remains inside *Acanthamoeba castellanii* and expresses virulence genes

**Figure 1:**
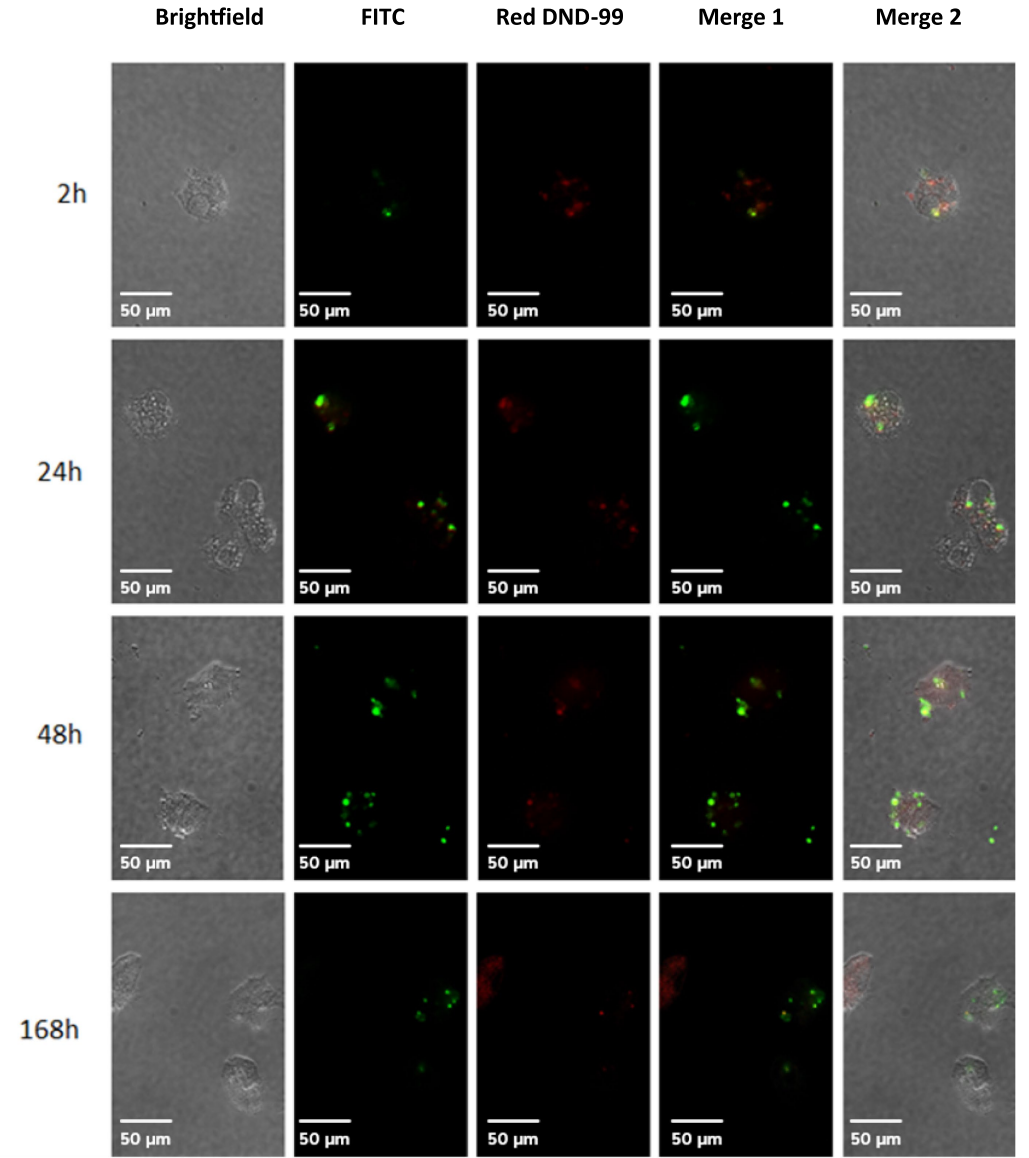
Infection kinetics of *Acanthamoeba castellanii* with *Piscirickettsia salmonis*. The infection kinetics were assessed at 2, 24, 48, and 168 h post-infection. *P. salmonis* was labeled with SRS Fluorotest (green), and *A. castellanii* lysosomes were stained with LysoTracker™ Red DND-99 (red). Scale bar: 50 μm.

**Figure 2:**
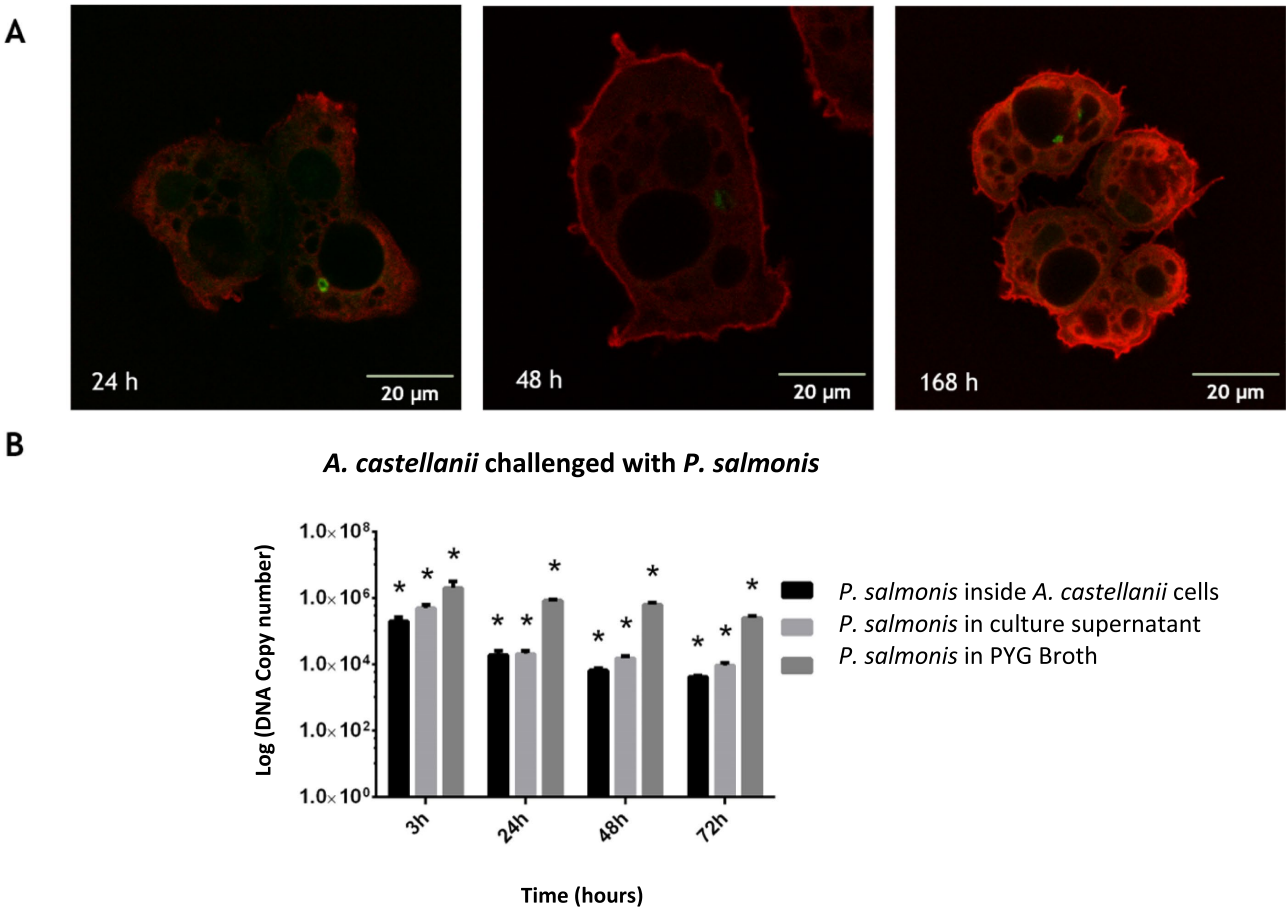
*P. salmonis* is maintained within digestive vacuoles of *A. castellanii* during in vitro infection. **(A)** Infection kinetics at 24, 48, and 168 h. *A. castellanii* was stained with LysoTracker™ Red DND-99 (red), and *P. salmonis* with Direct Fluorotest, Ango (green). The pathogen is present within amoebae at 24, 48, and 168 h, either within digestive vacuoles (phagosomes) or the contractile vacuole (168 h). Scale bar: 20 μm. **(B)** Quantification of *P. salmonis* within *A. castellanii* by qPCR. The number of bacteria was evaluated within the amoebae and in the supernatant at 3, 24, 48, and 72 h. ANOVA validation (*p < 0.05).

**Figure 3:**
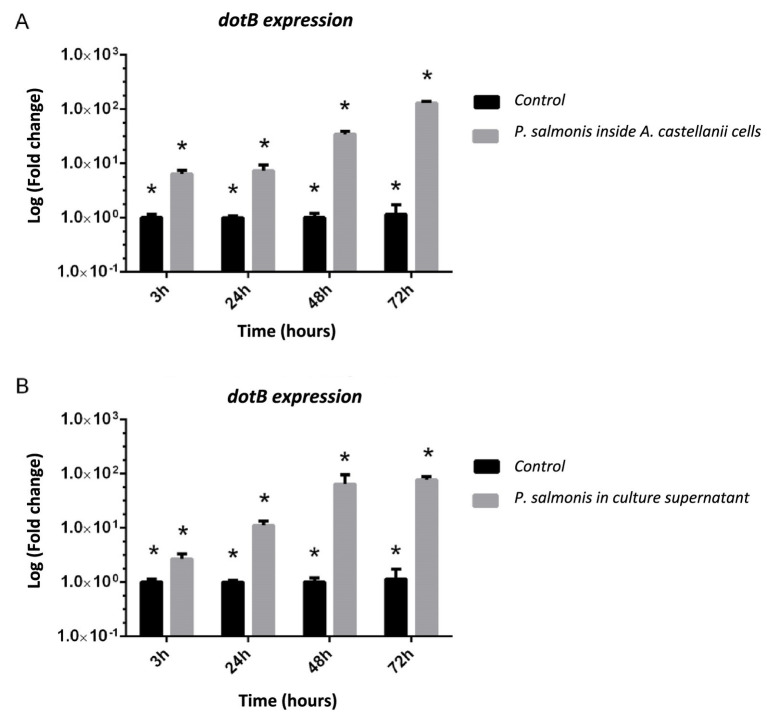
Relative expression of *P. salmonis dotB* gene (Dot/Icm Secretion System) during infection of *A. castellanii*. **(A)** *dotB* gene expression of *P. salmonis* inside *A. castellanii* cells at 3, 24, 48, and 72 h post-infection. **(B)** *dotB* gene expression of *P. salmonis* from supernatants of *A. castellanii* infected cultures at 3, 24, 48, and 72 h. ANOVA validation statistical significance was assessed by one-way ANOVA (*p < 0.05). In all cases bars represent mean ± SD from three independent biological replicates (*n* = 3).

*Acanthamoeba castellanii* cultures were challenged with *P. salmonis* and analyzed to assess infection kinetics at 2, 24, 48, and 168 h post-infection. Figure 1 shows that at different time points post-infection, *P. salmonis* is found within the digestive vacuoles of *A. castellanii*, indicating that the bacterium is capable of infecting and being internally processed by the amoeba. Confocal microscopy revealed that *P. salmonis* is maintained in both digestive and contractile vacuoles of *A. castellanii* for up to 7 days (168 h), as observed at different times post-infection (Figure 2A). This result suggests that the bacterium can resist digestion by the amoeba and maintain itself over time. Quantification of the bacterial load in infected *A. castellanii* cultures showed a decrease in *P. salmonis* from approximately 10^5 at 2 h to 10^4 at 72 h (Figure 2B). Figure 3 shows the expression profiles of the *dotB* gene, encoding a component of the Dot/Icm secretion system crucial for the intracellular survival of *P. salmonis* in macrophages. A marked overexpression of *dotB* is observed during infection in amoebae, with up to a 10^2-fold increase compared to the control at the final infection time point.

### *Acanthamoeba castellanii* acts as a vector for *Piscirickettsia salmonis in vitro*

**Figure 4:**
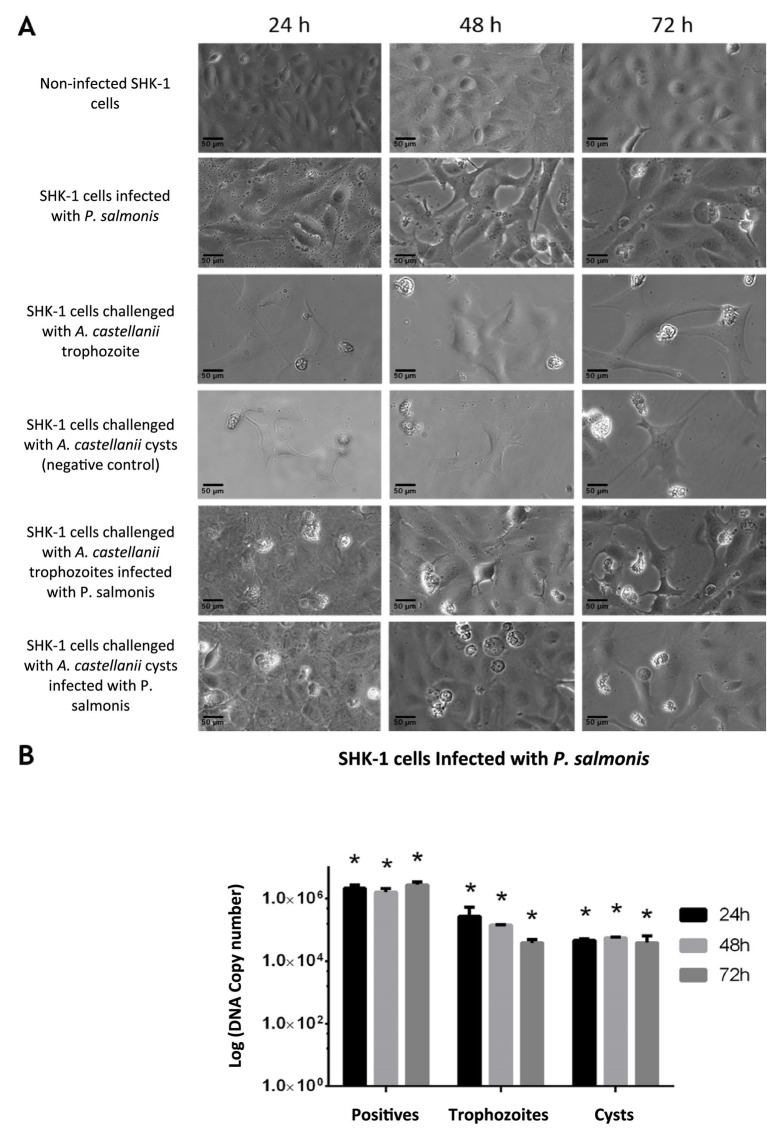
Challenge of SHK-1 cells with *A. castellanii* previously infected with *P. salmonis*. **(A)** Bright-field microscopy of SHK-1 cells challenged with *A. castellanii* infected with *P. salmonis* at 24, 48 and 72 h post-infection, showing cytopathic effects (Scale bar: 50 μm). **(B)** Quantification of *P. salmonis* in challenged SHK-1 cells by qPCR. The number of bacteria was evaluated within the cells and in the supernatant at 24, 48, and 72 h. ANOVA validation (*p < 0.05).

The ability of *A. castellanii* to act as a vector for the transmission of *P. salmonis* under *in vitro* conditions was evaluated. A kinetic study was performed in which SHK-1 cells (*S. salar* macrophages) were challenged with *A. castellanii* previously infected with *P. salmonis*. The progress of the infection was monitored microscopically until the appearance of the classic cytopathic effect (CPE) associated with *P. salmonis* infection. Results show that in SHK-1 cells challenged with trophozoites and cysts of *A. castellanii* infected with the bacterium, the typical CPE caused by *P. salmonis* was observed 72 h post-challenge, characterized by vacuolated and shiny cells (Figure 3A). Furthermore, free bacteria were detected in the supernatant of the challenged cell cultures, and the bacteria were detected in the SHK-1 cells by qPCR (Figure 4A). Quantitative analyses carried out by qPCR reinforced these observations, indicating bacteria were present in the medium (Figure 4B).

### Isolation of marine amoeba from fish gills

**Figure 5:**
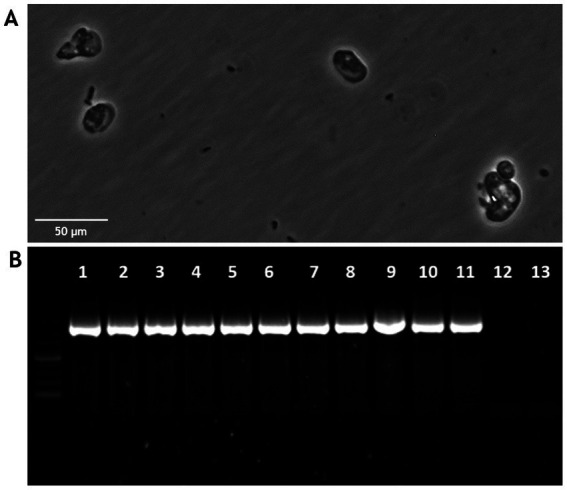
Isolation of marine amoeba *Vannella* sp. from the gills of *Salmo salar*. **(A)** Morphological verification of the isolated *Vannella* sp., according to Smirnov et al. (2007). **(B)** Verification of *Vannella* sp. by PCR targeting 18S rRNA. A 1200 bp amplicon of the expected size was observed. 1–11: different cultures of isolated amoeba; 12: negative control (DNA *P. salmonis* EM-90); 13: PCR blank. Was use a 100 bp Plus DNA Ladder (Thermo Fisher).

The marine protozoan was successfully isolated from the gills of *S. salar* specimens at a culture center experiencing an active outbreak of SRS in the Region of Los Lagos, exhibiting clinical signs of gill amoebiasis. After 15 days of transfers and washes to eliminate excess contaminating bacteria, a culture was obtained containing protozoa of similar morphology, which adhered to the bottom of the plate. Based on morphology and analysis by PCR using primers directed at the 18S rRNA gene, the protozoan was identified as an organism of the genus *Vannella* (Figure 5).

### Detection of symbiotic bacteria and *Piscirickettsia salmonis* in *Vannella* sp

**Figure 6:**
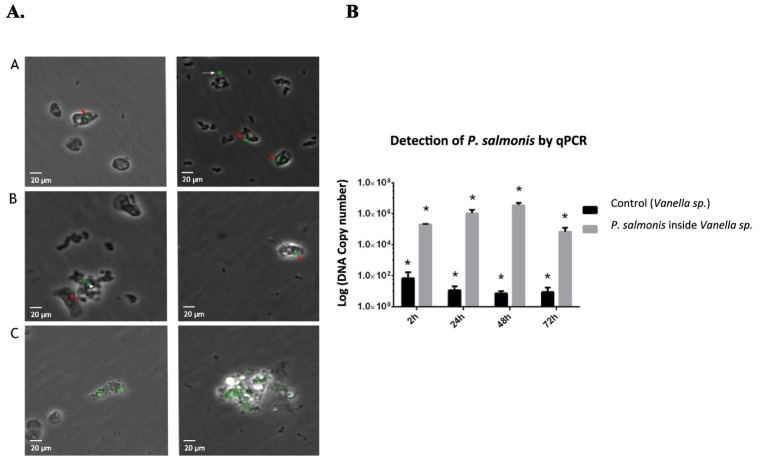
Marine amoeba *Vannella* sp. isolated from gills naturally contains *P. salmonis*. **(A)** IFAT of *P. salmonis* (green) in non-challenged *Vannella* sp. Red arrows indicate *P. salmonis* within the protozoan. White arrows indicate the presence of *P. salmonis* in the external medium (part of a biofilm). **(B)** Quantification of *P. salmonis* in *Vannella* sp. by qPCR. Number of DNA copies in reference to ITS amplifications. The gray bar corresponds to protozoa challenged with *P. salmonis*, and the black bar to non-challenged (untreated) protozoa. ANOVA validation (*p < 0.05).

In the evaluation of internal bacteria present in impure cultures of *Vannella* sp., the use of different antibiotics (streptomycin, gentamicin, kanamycin, Polymyxin B, and ciprofloxacin), either alone or in combination, was ineffective in obtaining pure cultures, demonstrating the symbiotic bacteria were resistant to these antibiotics. However, imipenem at all concentrations tested (3 μg/mL to 30 μg/mL) allowed for the elimination of bacterial content from the extracellular medium. Yet, after 48 h of removing the antibiotic from the trophozoites, bacterial growth was again observed, unlike in the controls, suggesting that *Vannella* sp. harbors these bacteria in digestive vacuoles. Through 16S bacterial sequencing, the predominantly present bacteria in *Vannella* sp. were identified as belonging to the genera *Alcanivorax* and *Vibrio*. Additionally, immunofluorescence detected *P. salmonis* inside *Vannella* sp. isolated from fish gills, both in the external medium and within biofilms or bacterial aggregates associated with the amoeba (Figure 6A). These results were confirmed by qPCR, which quantified the load of *P. salmonis* within *Vannella* sp., which was low but sufficient to be detected by IFAT and qPCR (Figure 6B).

### *Demonstration that Piscirickettsia salmonis* can infect *Vannella* sp. isolated from fish gills

**Figure 7:**
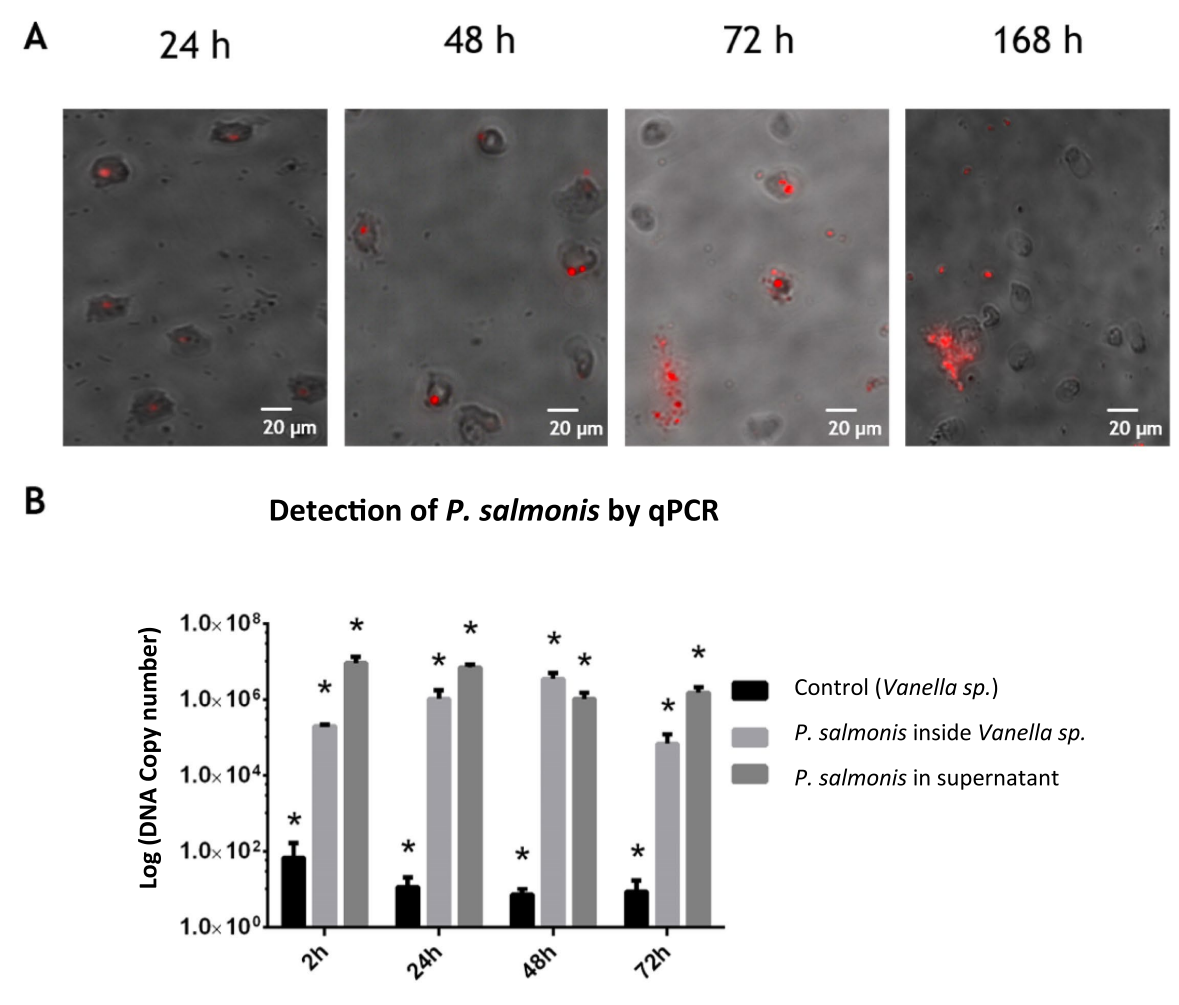
Infection kinetics of *Vannella* sp. with *P. salmonis in vitro*. **(A)** Immunofluorescence of the kinetics conducted at 24, 48, and 168 h post-challenge. *P. salmonis* was marked with R18 membrane staining (Invitrogen), and *Vannella* sp. is unmarked. Internalization of the pathogen was observed up to 168 h, with amoebic cellular lysis starting at 72 h. Scale bar: 20 μm. **(B)** Quantification of *P. salmonis* in infected amoebas. The black bar represents non-challenged (untreated) protozoa, the light gray bar represents protozoa challenged with *P. salmonis*, and the dark gray bar represents *P. salmonis* in the supernatant. Each data point represents mean ± SD from three independent experiments (*n* = 3). ANOVA validation (*p* < 0.05).

*In vitro* infection kinetics demonstrated that *P. salmonis* is capable of infecting and persisting within *Vannella* sp. for at least 168 h (7 days). However, starting at 72 h post-challenge, cellular lysis of the amoebae by the pathogen was observed, a phenomenon not seen in *A. castellanii* (Figure 7A), suggesting that *P. salmonis* may generate a productive infection in *Vannella* sp. These findings are supported by the results quantifying the number of *P. salmonis* units within *Vannella* sp. by qPCR, which increased at least until 48 h post-infection, reaching 10^7, and declined to 10^5 by 72 h (Figure 7B). Similarly, the number of *P. salmonis* units present in the supernatant of infected *Vannella* sp. cultures decreased after 48 h, coinciding with an increase in the internal quantity of *P. salmonis* units. Conversely, a slight increase of the pathogen in the supernatant and a decrease in its presence within the protozoan was observed at 72 h. Given that the behavior of the infection process has been similar to that observed in fish cells, the expression of structural genes of the Type IV-B Dot/Icm secretion system of *P. salmonis* was evaluated. These genes, used by the bacteria to replicate within fish macrophages, showed greater expression at early infection times, similar to that observed in the SHK-1 cell line.

### *Vannella* sp. acts as a vector for *Piscirickettsia salmonis in vivo*

**Figure 8:**
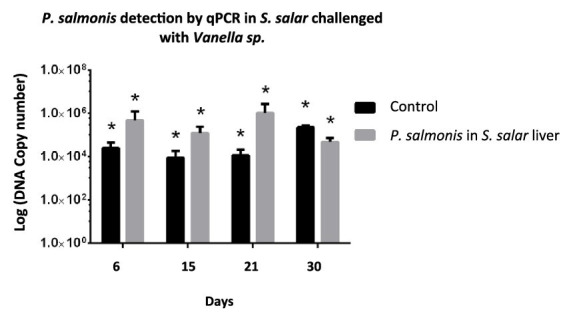
Quantification of *P. salmonis* in the liver of *S. salar* specimens challenged with *Vannella* sp. infected with the pathogen. The number of bacteria was evaluated by qPCR on days 6, 15, 21, and 30 post-challenge. ANOVA validation was performed (*p* < 0.05). The black line represents the control (*S. salar* injected intraperitoneally with *P. salmonis*), while the gray line indicates *P. salmonis* units in the liver of *Salmo salar* challenged with *Vannella* sp. infected with the pathogen.

An *in vivo* assay was conducted under controlled conditions to evaluate the ability of *Vannella* sp. to act as a vector for *P. salmonis*. The results demonstrated that the marine amoeba previously infected with *P. salmonis* can act as a vector for the pathogen toward *S. salar* specimens. The liver of each fish exposed to the presence of infected *Vannella* sp. was analyzed to determine *P. salmonis* as the causal agent of infection. The bacteria were present in the organs of the fish for at least 31 days, reaching a maximum order of 10^6 bacteria in the tissue, surpassing the control by 21 days (Figure 8). However, despite detecting its presence, no deaths of the infected fish were observed during the assessment periods; only typical signs of SRS were noted in the extracted liver.

## Discussion

*Piscirickettsia salmonis* remains a significant threat to the sustainability of salmon farming in Chile. This pathogen is difficult to culture in conventional media but can be propagated under enriched axenic conditions (Henríquez et al., 2013). Nevertheless, *P. salmonis* persists in the marine environment and can be transmitted between both salmonids and non-salmonids (Contreras-Lynch et al., 2017; Schober et al., 2023). The shedding of infectious bacteria from infected fish does not fully account for the extent of outbreaks affecting net pens near harvest, which often results in substantial economic losses.

In this context, it has been extensively reported that pathogenic bacteria in aquatic environments frequently establish symbiotic relationships with free-living protozoa (FLA, or free-living amoebae). These interactions enable bacteria to develop various survival strategies, including persistence and replication within FLA (Schuster and Visvesvara, 2004; Balczun and Scheid, 2017; Scheid, 2018; Fan et al., 2024).

The symbiotic relationship between *Acanthamoeba* and its bacterial endosymbionts is a critical factor in the pathophysiology of these ubiquitous protists. In fact, *Acanthamoeba* serves as an evolutionary “melting pot,” fostering an environment rich in genetic exchange between co-inhabiting bacteria, a process particularly evident among Rickettsiales endosymbionts (Wang and Wu, 2017). This distinctive intracellular domain facilitates extensive lateral gene transfer (LGT), a mechanism through which endosymbionts acquire genes crucial for their survival and interaction within the amoeba host, favoring among others, enhanced bacterial virulence, adaptation to intracellular lifestyles increased antibiotic resistance, and robust stress responses, contributing to the endosymbionts’ pathogenic potential and their capacity to cause disease in human and animal hosts. As an example, recent studies demonstrate that the presence of intracellular bacteria in *Acanthamoeba* isolates from human corneal infections have exacerbated its pathogenicity enhancing the severity of Acanthamoeba keratitis (Rayamajhee et al., 2024; Rayamajhee et al., 2025). These endosymbionts, particularly gram-negative bacteria or *Chlamydia* -like organisms, enhance the amoeba’s virulence by inducing quicker cytopathic effects on host cells (Fritsche et al., 1998). Furthermore, in experimental models, the acquisition of bacteria like *Pseudomonas aeruginosa* by *Acanthamoeba* markedly increases the severity of the infection, suggesting that the simultaneous presence and potential release of these intracellular bacteria significantly contribute to the progression of the disease (Rayamajhee et al., 2024).

Consequently, FLA may act as vectors or “Trojan horses,” facilitating pathogen spread to various hosts, particularly in confined and stressed environments such as salmonid net pens near harvest. Understanding how *P. salmonis* infects healthy salmonids in the marine environment is crucial; this research aimed to investigate a potential relationship between the bacterium and free-living amoebae, organisms known to inhabit net pen environments, as a potential survival and dissemination strategy. It is important to clarify that, in our *in vivo* experimental design, only the positive control group (Tank 1) was injected intraperitoneally with *P. salmonis*. In contrast, Tanks 2 and 3 corresponded to cohabitation assays, where fish were not injected but instead exposed to water containing *Vannella* sp. previously infected with *P. salmonis*. This approach was intended to simulate a more natural transmission scenario through contact with infected amoebae and to test whether the protozoa could act as a biological vector under aquatic conditions. The detection of *P. salmonis* in the liver of fish from these cohabitation tanks provides experimental support for the potential role of free-living amoebae as reservoirs and vehicles for pathogen persistence in the marine environment.

Our hypothesis posits that exposure to environmental stressors and drastic changes in seawater conditions drive *P. salmonis* to seek refuge in more stable environments to enhance its persistence, replication, and transmission. Has been described that biofilm formation represents a key ecological strategy that enhances the survival and persistence of *P. salmonis* in marine environments. Early observations by Marshall et al. (1998) demonstrated the bacterium’s capacity to produce biofilm-like structures, suggesting a mechanism that supports its survival outside host cells. Subsequent studies have confirmed that *P. salmonis* can develop mature and resilient biofilms on abiotic surfaces exposed to seawater, which remain stable under environmental stress and in the presence of salmon skin mucus (Levipán et al., 2020; Santibañez et al., 2020). These findings strengthen the notion that biofilm formation, together with interactions involving protozoan hosts, constitutes a complementary strategy that promotes environmental persistence and may facilitate the transmission of *P. salmonis* within aquaculture systems. Recent experimental evidence indicates that *P. salmonis* employs complex mechanisms and active metabolic pathways essential for its adaptability to environmental stress (Schober et al., 2023; Aliaga-Tobar et al., 2025). Furthermore, interactions between amoebae and bacteria can range from mutualistic to parasitic (Ali et al., 2024). The genetic complexity and versatility of *P. salmonis* likely favor the manipulation of this relationship for its own benefit. The two FLA species analyzed live in both fresh and salt water, suggesting that the observed symbiosis could also involve the early stages of salmonid fish grown in confinement, when the bacteria is detectable even without clinical signs. In summary, our results suggest that protozoa can serve as environmental reservoirs and potential vectors for *P. salmonis*, enhancing our understanding of the pathogen’s life cycle and informing the development of novel disease control strategies. Additionally, these findings imply that FLA may represent a stable reservoir and a source of confinement and dissemination for other pathogens associated with marine aquaculture.

Our *in vivo* results support the feasibility of the amoeba-mediated transmission hypothesis under controlled experimental conditions. The detection of *P. salmonis* within amoebae, followed by its successful recovery from infected fish, demonstrates that the bacterium can remain viable and infectious after residing intracellularly. The absence of mortality in fish exposed to *Vannella*-borne *P. salmonis* is consistent with a low-dose or subclinical infection, as expected for amoeba-mediated transfer. While the detection of bacterial DNA in liver confirms systemic infection, these findings demonstrate viability rather than confirmed horizontal transmission. Nevertheless, these results do not provide direct evidence of horizontal transmission under natural marine conditions. Further validation through cohabitation experiments, field surveys, and epidemiological monitoring will be necessary to determine whether amoebae act as genuine vectors or merely as transient environmental hosts of *P. salmonis*.

Finally, our results also demonstrate that free-living amoebae can internalize and maintain *P. salmonis*, suggesting that these protozoa may serve as environmental reservoirs that contribute to the persistence of the pathogen in aquatic systems. In our experiments, although intraperitoneal injection was used only as a positive control, the immersion exposure with amoebae better approximates natural environmental contact. Cohabitation and Trojan-fish challenges remain valuable approaches for future validation of amoeba-mediated transmission under near-field conditions. This ecological perspective may help improving future prevention strategies for Salmonid Rickettsial Septicemia.

## Data Availability

The original contributions presented in the study are included in the article/Supplementary material, further inquiries can be directed to the corresponding author/s.
